# Woven EndoBridge embolization in the retreatment of basilar apex aneurysm

**DOI:** 10.3171/2022.7.FOCVID21152

**Published:** 2022-10-01

**Authors:** Jae Eun Lee, Visish M. Srinivasan, Peter Kan

**Affiliations:** 1 Department of Neurosurgery, Baylor College of Medicine, Houston, Texas;; 2 Department of Neurosurgery, Barrow Neurological Institute, Phoenix, Arizona; and; 3 Department of Neurosurgery, University of Texas Medical Branch, Galveston, Texas

**Keywords:** neurosurgery, endovascular, aneurysm

## Abstract

The Woven EndoBridge (WEB) device was approved by the U.S. FDA for its excellent angiographic occlusion of intracranial aneurysms and high safety profile, based on the landmark WEB-IT (WEB Intrasaccular Therapy) trial. There remains, however, a few cases of aneurysm recurrence that necessitate retreatment after the initial WEB procedure. In this technical video, the authors present the case of a middle-aged patient who showed significant basilar apex aneurysm recurrence and growth along with device compaction that required retreatment. Various aspects of treating aneurysms with a prior WEB device, including procedural technique, are discussed.

The video can be found here: https://stream.cadmore.media/r10.3171/2022.7.FOCVID21152

## Transcript

We present WEB embolization in the retreatment of a basilar apex aneurysm.

### 0:27 Patient Presentation.

The patient was a 60-year-old man who underwent treatment of his unruptured basilar apex aneurysm with the WEB SL device, sized 7 × 5 mm.^1–3^ Note the oversizing of the device relative to the aneurysm dimensions. At the 12-month follow-up, he was noted to have significant recurrence of aneurysm with growth around the base of the aneurysm, with compaction of the device. He remained neurologically intact on follow-up.

### 0:53 Retreatment Planning.

After discussion with the patient and informed consent, we proceeded with retreatment of the aneurysm. Our setup consisted of a 6-Fr slender sheath in the right radial artery, through which a Benchmark guide catheter was inserted. A Penumbra select catheter was used to access the vertebral artery. From there, a Sofia 5-Fr intermediate catheter and Via 21 catheter were used to access the aneurysm and deploy a new WEB SL device.

### 1:19 Initial Access and Baseline Imaging.

The right vertebral artery is being accessed here with the Penumbra select catheter over a 0.035 Glidewire. The Benchmark guide catheter is then advanced into the mid-V2 segment. These baseline cranial runs demonstrate the recurrent basilar apex aneurysm. The 3D rotational angiogram demonstrates the anatomy at the basilar apex, with the aneurysm projecting superiorly and slightly posteriorly. The markers of the initial WEB device are seen in the upper left recess of the aneurysm, consistent with aneurysm recurrence at the base and device compaction. A magnified AP and lateral treatment view is here, from which we measure for the device.

### 1:54 Aneurysm Catheterization.

The aneurysm is being microcatheterized here. The wire is centered in the aneurysm as much as possible to obtain an ideal position for WEB deployment. The distal access catheter is positioned just below, providing support. Note the markers of the old device.

### 2:26 Web Sizing Concepts.

There are several concepts to keep in mind when considering sizing of the WEB device. In general, oversizing is preferred in WEB device selection.^4^ In flow diversion, parent artery wall apposition is the goal. In WEB flow disruption, aneurysm neck apposition is the goal. This is achieved by oversizing with lateral compression of the device. In this particular case, undersizing the device was most likely associated with the recurrence. Thus, in the retreatment, we tried to oversize for the recurrence. Because the aneurysm recurrence was smaller than the original aneurysm, a similar sized device was chosen. The recurrence rate of aneurysms treated by coil embolization is lower in smaller-sized aneurysm. However, with the WEB device, there is particular importance to sizing even for small-sized aneurysms. It is important to cover the complete neck and achieve aneurysm neck apposition because this sealing of the inflow results in elimination of the flow into the distal aspect of the aneurysm dome. This can result in quick stagnation. Thus, the WEB device does not have to accommodate the full size of the aneurysm, but rather may focus just on the neck, or the primary part of the aneurysm.

### 3:44 Device Deployment and Adjustment.

The new WEB device is then advanced in the microcatheter and deployed. The microcatheter position is slightly adjusted during deployment. The device opens, but in a suboptimal position low in the bifurcation. It is recaptured and resheathed. The second attempt at deployment is much better, straddling the neck well in the AP view.

### 4:05 Postdeployment.

In the postdeployment angiogram, note the contrast stasis on the posterior wall. The seal of the neck is much better now. The device is detached now. The lateral view demonstrates the WEB on WEB quite well. The contrast still hangs in the aneurysm several minutes later, as seen in this cone-beam CT in the sagittal direction.

### 4:33 Clinical Outcome.

At 6-month angiographic follow-up, the aneurysm is occluded and only a small neck remnant remains. The patient continues to be clinically asymptomatic. Thank you.

## Disclosures

The authors report no conflict of interest concerning the materials or methods used in this study or the findings specified in this publication.

## Author Contributions

Primary surgeon: Kan. Editing and drafting the video and abstract: all authors. Critically revising the work: all authors. Reviewed submitted version of the work: all authors. Approved the final version of the work on behalf of all authors: Kan. Supervision: Kan, Srinivasan.
